# The inactive X chromosome as a female protector in autism and beyond

**DOI:** 10.21203/rs.3.rs-7539254/v1

**Published:** 2025-09-08

**Authors:** Maya Talukdar, David C. Page

**Affiliations:** 1 Whitehead Institute, Cambridge, MA, USA; 2 Harvard/MIT MD-PhD Program, Program in Biomedical Informatics, Boston, MA, USA; 3 Department of Biology, Massachusetts Institute of Technology, Cambridge, MA 02139, USA; 4 Howard Hughes Medical Institute, Whitehead Institute, Cambridge, MA, USA

**Keywords:** female protective effect, autism, developmental disorders, sex differences, sex chromosomes

## Abstract

Why do human females require more autosomal genetic risk to manifest autism compared to males? Why is this also true of other male-biased, childhood-onset conditions with substantial autosomal heritability? Here we propose that the higher female liability threshold for these disorders arises due to gene expression from the human inactive X chromosome (Xi), which buffers the effects of deleterious autosomal variants. In contrast, we posit that gene expression from the Y chromosome is less effective than Xi at mitigating the consequences of such pathogenic autosomal alleles, contributing to the lower liability threshold observed in males. This framework unites epidemiological, genetic, and mechanistic observations across autism. Moreover, via a systematic review of 101 published genetic studies of male-biased conditions, we identify 16 other childhood disorders that empirically demonstrate an FPE – suggesting that Xi’s genetic activity throughout the body enables females to better tolerate autosomal genetic risk for a panoply of pediatric conditions. If correct, this reshapes our understanding of sex differences in disease and highlights the roles of Xi and the Y chromosome in mitigating or enhancing the effects of autosomally mediated liability.

## Introduction

Autism is a common, highly heritable^1–3^ neurodevelopmental condition characterized by difficulties with social communication, as well as restricted or repetitive behaviors and interests^4,5^. Autism is diagnosed in males about four times as often as in females^6–8^. This male bias is consistent across cultures, diagnostic criteria, and decades of epidemiological study^9^. While X-linked causes of autism and the underdiagnosis of autistic females contribute to this sex bias, they cannot fully explain it (**Supplementary Note**)^10^. Thus, the origins of autism’s male bias remain an open area of inquiry.

In the last twenty years, large-scale DNA sequencing studies of autistic individuals have consistently shown that, as a group, autistic females carry a higher burden of autosomal variation associated with autism than autistic males. This is true not only for rare, frequently *de novo* single-nucleotide variants (SNVs) and copy number variants (CNVs)^11–16^, but also for inherited recessive variants^17^; non-coding variants^18^; and common polygenic variants^15,19^. This increased autosomal genetic load for autism in diagnosed females compared to diagnosed males holds even when individuals are matched on traits such as autism severity or IQ, suggesting that this observation is not driven by ascertainment bias for more profoundly affected females^10,16,20,21^. Rather, it implies that females are somehow shielded from autosomal variants associated with autism, as they require more autosomal genetic risk to be diagnosed with autism compared to males. This phenomenon has been termed the “female protective effect” (FPE). Despite much interest in the FPE in autism, its underlying mechanisms remain unknown. In this Perspective, we argue that the human “inactive” X chromosome (Xi), traditionally viewed as transcriptionally silent, plays an active protective role in females by buffering the effects of pathogenic autosomal variants. This mechanism offers a unifying explanation for an elevated liability threshold in females and a consistent male bias not only in autism, but across a spectrum of childhood-onset conditions that display substantial autosomal heritability.

## A persistent puzzle: a higher liability threshold in females

The FPE in autism is most commonly conceptualized in terms of the “sex-differential liability threshold model” (SD-LTM)^10,22^. Of note, these terms, as well as “sex bias” or “male bias,” have been employed in varying and occasionally conflicting ways in the literature^10,22^. In the interest of clarity, we define these terms in Table 1. Originally proposed by Pearson and Lee in 1901^23^, the “liability threshold model” (LTM) assumes a population-wide, normal distribution of “liability” that consists of the sum of genetic and environmental factors underlying dichotomous diagnoses (*e.g*., whether or not an individual is diagnosed with autism). Under the LTM, individuals whose personal liability exceeds a single, population-wide threshold are diagnosed with autism. The SD-LTM, proposed by Carter in the 1960s^24,25^, extends the LTM by proposing that polygenic, sex-biased diseases exhibit sex-differential liability thresholds. Under the SD-LTM, then, the male bias of autism arises because females require a higher degree of liability to manifest autism compared to males.

Studies of sex differences in autism liability have borne out key predictions of Carter’s SD-LTM. First, as discussed earlier, autistic females harbor a greater genetic load of autism-associated variants, a measurable proxy for autism liability, than autistic males^11–17,19,10,20,21^. Second, the SD-LTM predicts that, on average, females without autism will exhibit higher autism liability than males without autism. Testing this proposition is challenging as it requires sequencing data from large cohorts of individuals from the general population. Nonetheless, there is compelling preliminary evidence that females in the general population harbor a modestly higher burden of autism-associated CNVs than do males^12,26–29^. The SD-LTM has also been supported by parent-of-origin analyses of inherited variants associated with autism. If females are genetically protected from autism compared to males, we would expect that inherited autism variants would more frequently be inherited from unaffected mothers than from unaffected fathers; mothers would be more likely than fathers to be unaffected carriers of these variants. Family-based studies have consistently shown this to be the case^12,30–33^.

Finally, the SD-LTM predicts that a more strongly male-biased sex ratio would be observed among autistic individuals with mutations in genes that confer moderate liability to autism (which may only exceed the male liability threshold) compared to those with mutations in high-risk autism genes (which confer larger increases in autism liability that may exceed both the male and female liability thresholds). Indeed, a recent study found a sex ratio of 4M:1F among individuals with mutations in moderate-risk genes, and a more modest sex ratio of 1.6M:1F among those with mutations in high-risk autism genes^34^.

**Figure 1.**
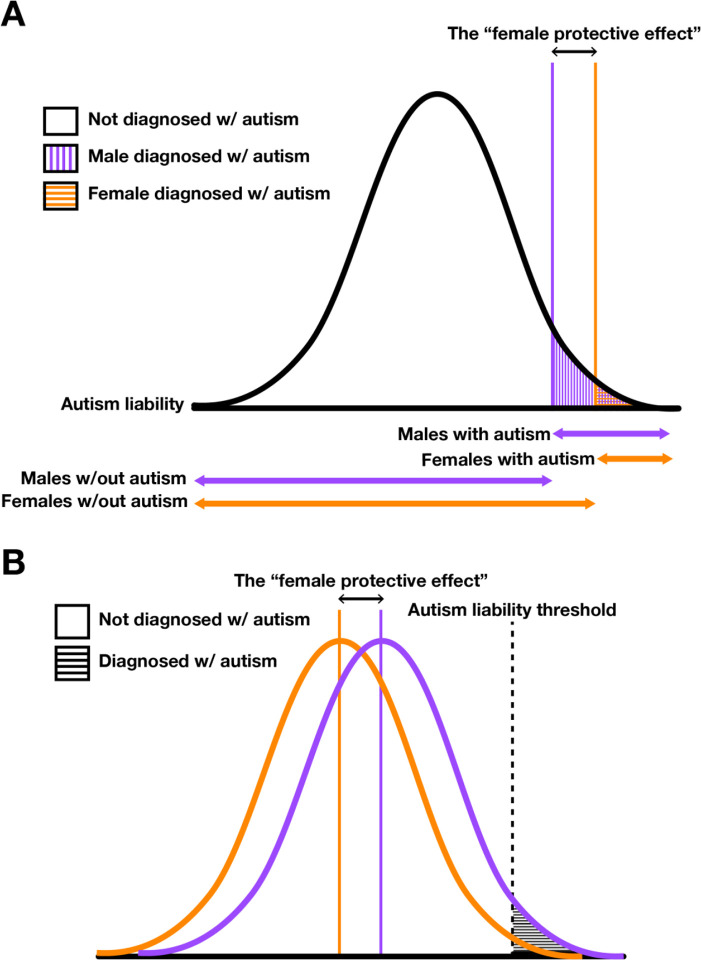
Schematics of the (A) single-distribution, and (B) double-distribution sex-differential liability threshold models (SD-LTMs). Orange represents female and purple represents male. The two models are quantitatively equivalent in that they can be parameterized to model the same male bias in autism prevalence. (**A**) In the single-distribution model, this bias arises from a mean liability threshold difference (ΔT) between males and females. (**B**) In the double-distribution model, this bias arises from a shift in overall mean liability (Δμ) between males and females. When ΔT = Δμ, the two models yield identical estimates for autism’s male bias. However, these models differ in their assumptions: the single-distribution model assumes a shared genetic liability distribution for autism with sex-specific thresholds, whereas the double-distribution model assumes that males and females have inherently different autism liability distributions.

While the SD-LTM provides a useful conceptual framing of the FPE in autism, it does not identity the biological basis of protection. Some investigators have hypothesized that males and females share a single liability threshold but that individual autism-associated variants increase liability more in males than females (Fig. 1A). While this is theoretically possible, sequencing studies of large cohorts of autistic individuals (n >10,000) have found no compelling evidence of widespread gene-by-sex effects^14,16,35^. This suggests that the male bias and FPE in autism are not primarily driven by variants with sex-differential effects on autism liability – rather, it implies a generally decreased susceptibility of females to autosomal autism-associated variation compared to males (Fig. 1B).

## The sex chromosomes modify autosomal liability in autism

Various factors have been proposed to explain the lower susceptibility to autism in females as compared to males, including imprinting, sex hormones, and sex differences in baseline cognitive-behavioral traits^10^. However, no definitive explanation for the FPE has emerged. We posit that the lower susceptibility of females to autism is due to fundamental genetic differences between the 46^th^ chromosomes in female and male human somatic cells: the inactive X (Xi) in females, and Chr Y in males. The elements of our proposal are as follows:

1. We will argue here that sex differences in autism susceptibility arise from functional differences between Xi and Chr Y in human somatic cells. In these cells, Chr X exists in one of two epigenetic states: the so-called “active” configuration, or Xa, and the “inactive” configuration, or Xi. The cells of typical XX females have one Xa and one Xi, whereas the cells of typical XY males have one Xa and one Chr Y. Our laboratory has recently reported evidence that the Xa chromosomes found in XX females and XY males are functionally and epigenetically equivalent^36,37^. Thus, sex differences should arise from differences in the genetic activities of Xi and Chr Y.
2. Despite its “inactive” moniker, it is known that the human Xi expresses at least 60 different X-linked protein-coding genes^36^. These Xi-expressed genes are also transcribed from Xa. As a consequence, Xi-expressed genes exhibit higher transcript levels in XX females than in XY males.
3. Many of these Xi-expressed genes encode global regulators of core molecular processes (*e.g.*, chromatin modification and transcription) that engage thousands of autosomal genes. These core molecular processes, and the autosomal genes they employ, are frequently disrupted in autism^38^.
4. Combining these insights, *we hypothesize that the FPE arises because females are less susceptible to disruptions in core molecular processes that are regulated by Xi-expressed genes, including disruptions caused by mutations in autosomal autism risk genes. This protective effect is due in part to the higher expression in females than males of genes expressed from Xi (and Xa)*. We emphasize that this is not due to sex-differential vulnerability to mutations on the sex chromosomes but rather to sex-differential vulnerability to disruptions in autosomal genes and pathways regulated by Xi-expressed genes. Put another way, we posit that Xi acts as a protective genetic modifier of autosomal autism liability in females.

We will now expand upon each of these four elements of our proposal.

### Differences between Xi and Chr Y drive biological differences between females and males

The X and Y chromosomes are transmitted from one generation to the next in a manner that distinguishes them from autosomes. Mothers transmit one of their two X chromosomes to daughters and sons alike, while fathers transmit their single X chromosome to daughters and their Y chromosome to sons. This familiar story of sex chromosome genetics is inextricably intertwined with the story of X chromosome inactivation (XCI). XCI serves as a dosage compensation method to balance the expression of X-linked genes between XX females and XY males^39^. During embryonic development, one of the two X chromosomes in each somatic cell of an XX female is randomly epigenetically inactivated. As a result, the two X chromosomes in typical XX females exist in two distinct epigenetic states: the aforementioned active X chromosome Xa and “inactive” X chromosome Xi. In contrast, somatic cells in XY males contain an Xa and a Chr Y.

Recent quantitative studies of protein-coding transcripts in human somatic cells *in vitro* and *in vivo* indicate that Xa chromosomes found in typical XX females are epigenetically indistinguishable from those found in typical XY males^36,37^. Thus, from the perspective of somatic physiology and function, Xa does not differentiate between females and males. Rather, Xa is effectively the 45^th^ autosome, shared by females and males. Biologically based sex differences, including sex differences in susceptibility to autosomal autism liability, should therefore trace their mechanistic origins to differences in the genetic activities of Xi and Chr Y – the 46^th^ chromosome.

### Despite its name, the “inactive” X chromosome is quite active genetically

Since Mary Lyon’s coining of the term in 1961^40^, “X chromosome inactivation” has been understood to mean genetic silencing of the second X chromosome in somatic cells of XX females. Nonetheless, beginning in the 1980s, a series of studies have provided overwhelming evidence that a subset of human X-linked protein-coding genes are transcribed from Xi in XX females^41^. Without exception, these Xi-expressed protein-coding genes are also transcribed from Xa in both XX females and XY males, so the total expression of these genes is greater in XX females than XY males. This set of genes has been theorized to drive broader phenotypic sex differences^42,43^. However, we have not fully understood which genes are expressed from Xi, their levels of Xi expression, and their biological functions in health and disease.

Building upon previous studies, our group recently demonstrated that more than 60 X-linked protein-coding genes are expressed from Xi^36^. These genes were identified by modeling changes in expression of X-linked genes in response to Xi copy number, leveraging a unique cohort of nearly two hundred individuals with sex chromosome aneuploidies (zero to three Xis and zero to four copies of Chr Y). Importantly, these 60 or so Xi-expressed genes are not a random subset of X-linked genes. Rather, many of them are widely expressed, dosage-sensitive genes that act as global regulators of processes like chromatin modification, transcription, and translation and thus influence the activity of thousands of autosomal genes^44,45^. Thus, female somatic tissues exhibit higher expression of many X-linked global regulators compared to male somatic tissues.

An immediate question that arises is whether genes on Chr Y compensate for this lower expression of these Xi-expressed global regulators in male somatic tissues. As it turns out, Chr Y carries only 27 protein-coding genes or gene families, including the testis-determining gene *SRY* and nine spermatogenesis factors expressed largely or exclusively in testicular germ cells^46^. Thus, in somatic cells, most X-linked, Xi-expressed genes have no active counterpart on Chr Y. Nonetheless, a dozen Chr Y genes encode broadly-expressed global regulators of key molecular processes, and all 12 have X-linked homologs expressed from both Xi and Xa^46,47^. Taken together then, Xi-expressed genes can be divided into those with and those without a counterpart on Chr Y: the former group will be referred to here as the X-Y gene pairs. Both of these classes of Xi-expressed genes are expressed at higher levels in females than in males, providing clues for explaining the FPE.

### Molecular processes disrupted in autism are modulated by Xi-expressed genes

**Figure 2.**
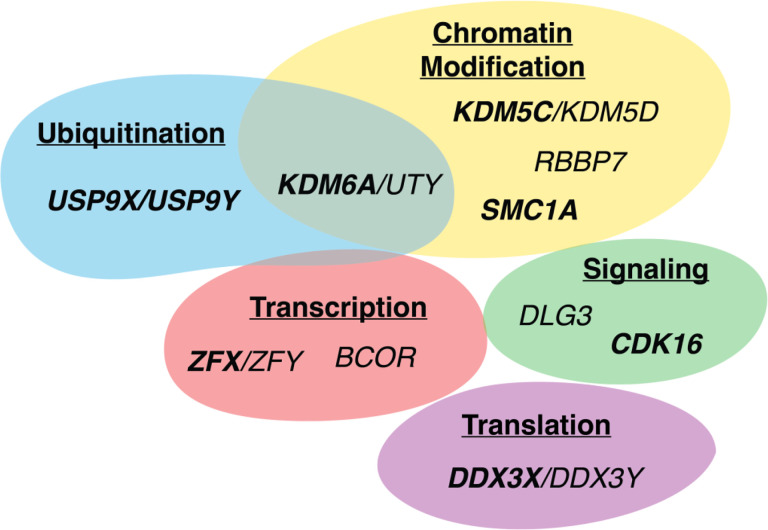
Cell biological functions of ten of the most highly dosage-sensitive, Xi-expressed genes (San Roman et al. 2023)^36^ and their Y-linked homologs. Genes implicated in autism (*SFARI Gene*) are bolded. Functions based on *UniProt*.

Numerous genetic studies have shown that autism-associated mutations disproportionately involve genes that are both widely-expressed and dosage-sensitive, and particularly those encoding global regulators^48,49^. Many Xi-expressed genes share these characteristics^36,45^, suggesting the possibility of a mechanistic link between Xi gene expression and autism risk. In fact, nearly one quarter of Xi-expressed genes have been directly implicated in autism, including seven of the ten most dosage-sensitive genes expressed from Xi^36^ (Fig. 2). These Xi-expressed genes govern central cellular activities like chromatin modification, splicing, and transcription (Fig. 2), which are frequently disrupted in autism^38,50^. For example, mutations in the Xi-expressed transcription factor *ZFX* cause a male-biased neurodevelopmental disorder with autistic traits^51^. We recently showed that *ZFX* is a key modulator of autism-associated transcriptional programs in human neural progenitor cells^52^. Taken together, these findings suggest that Xi-expressed are not merely associated with autism but regulate the core processes responsible for its development.

### How Xi may account for the female protective effect in autism

Based on these findings, we posit a mechanism for how Xi-expressed genes contribute to decreased female vulnerability to autism, drawing on the model put forth in San Roman et al. 2023^36^. They proposed that dosage-sensitive, Xi-expressed genes play a central role in driving biological sex differences as they exhibit female-biased expression (by virtue of being Xi-expressed) that is likely phenotypically consequential (by virtue of being dosage-sensitive). To understand how such Xi-expressed genes can shape sex differences in autism susceptibility, we first turn to the Xi-expressed protein-coding genes that have no Y-linked homologs. These “Xi-not-Y” genes are expressed at higher levels in females than males because females express them from both Xa and Xi, whereas males only express them from Xa, with no opportunity for compensation by a Y-linked homolog. We therefore hypothesize that female-biased expression of such dosage-sensitive, Xi-not-Y genes contributes to an FPE by allowing females to better buffer disruptions in central cellular processes, including those resulting from mutations in autosomal autism genes.

There is mounting evidence that X-Y gene pairs also contribute to the FPE in autism. Autism genes are strongly represented amongst these paired genes, with two (*NLGN4Y*, *USP9Y*) expressed from Chr Y, and seven (*DDX3X*, *KDM5C*, *KDM6A*, *NLGN4X*, *TBL1X*, *USP9X*, and *ZFX*) from Xi. Whether these X-Y gene pairs contribute to the FPE in autism will depend on the degree to which the X-linked and Y-linked homologs have diverged in function, expression, or regulation over the course of their independent evolution. Studies of *DDX3X* and *DDX3Y* provide an intriguing example. Xi-expressed gene *DDX3X* is among the most conistently associated risk genes for autism and intellectual disability^53,54^. In contrast, mutations in its Y-linked homolog *DDX3Y* are most frequently ascertained in healthy adult males presenting with infertility^55^ and have not been associated with autism. Future studies will determine how *ZFX*’s transcriptional regulation of autism risk genes contributes to the FPE in autism, based on comparing *ZFX* and *ZFY* in diverse *in vitro* and *in vivo* contexts.

## Beyond autism: a shared basis for other male-biased pediatric disorders

Although the FPE is primarily discussed in the context of autism, both the FPE and the SD-LTM lead to male biases in other disorders. The SD-LTM was first conceptualized by Carter in 1961 to describe the male bias of pyloric stenosis, a common congenital gastrointestinal anomaly that, like autism, affects about four males for every affected female^24,25^. Carter noted that mothers with pyloric stenosis were more likely than affected fathers to have an affected child – and especially an affected son. Carter additionally reported a higher recurrence rate of pyloric stenosis in families where females were affected than in families where only males were affected. Taken together, this suggests that affected females harbor more genetic liability for pyloric stenosis than affected males, consistent with pyloric stenosis exhibiting an FPE. The finding that familial recurrence rates are higher when the index case is a member of the less frequently affected sex, now known as “the Carter effect”, provides evidence that a sex-biased disorder follows an SD-LTM. Several other male-biased disorders, including clubfoot and congenital heart disease, were later shown to exhibit the Carter effect, suggesting that they also feature FPEs^56,57^. Indeed, a 2022 study found that the increased burden of deleterious *de novo* variants observed in autistic females compared to autistic males is driven solely by mutations in genes associated with *both* autism and developmental disorders – rather than genes implicated only in autism^58^. In other words, the FPE observed in autism may be a general feature of male-biased developmental and congenital disorders. We thus sought to test for such a general feature in other male-biased disorders.

**Figure 3.**
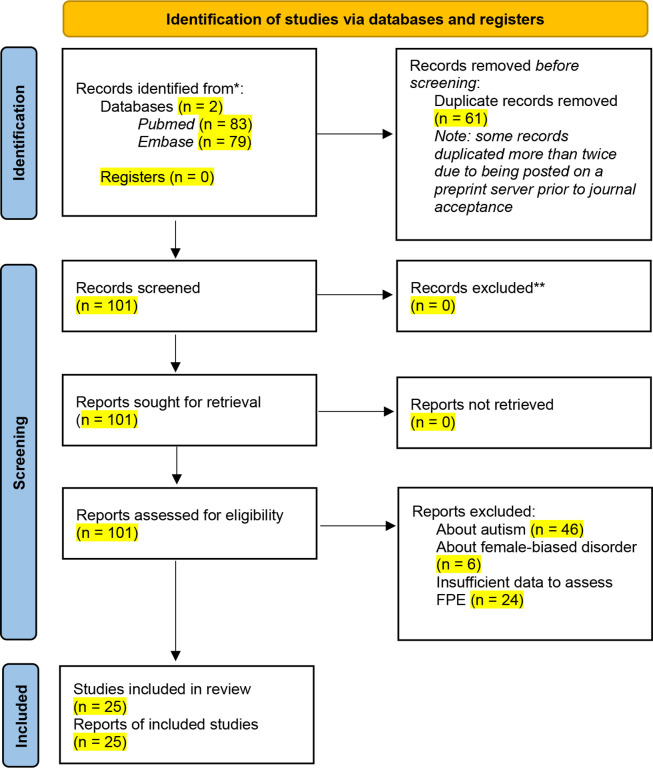
PRISMA diagram of strategy used for literature review of additional male-biased disorders beyond autism that empirically demonstrate an FPE. Associated with Table 2. 162 genetic studies (61 duplicates) of male-biased pediatric conditions were obtained from the PubMed and Embase databases for preliminary screening. Of these 101 unique studies, 46 were discluded for a primary topic of autism, 6 were discluded for a primary topic of a female-biased disorder (adolescent idiopathic scoliosis and multiple sclerosis), and 24 were excluded due to insufficient data to assess the presence of an FPE. From the 25 remaining studies, we report 16 male-biased disorders that empirically demonstrate an FPE in Fig. 4.

**Figure 4.**
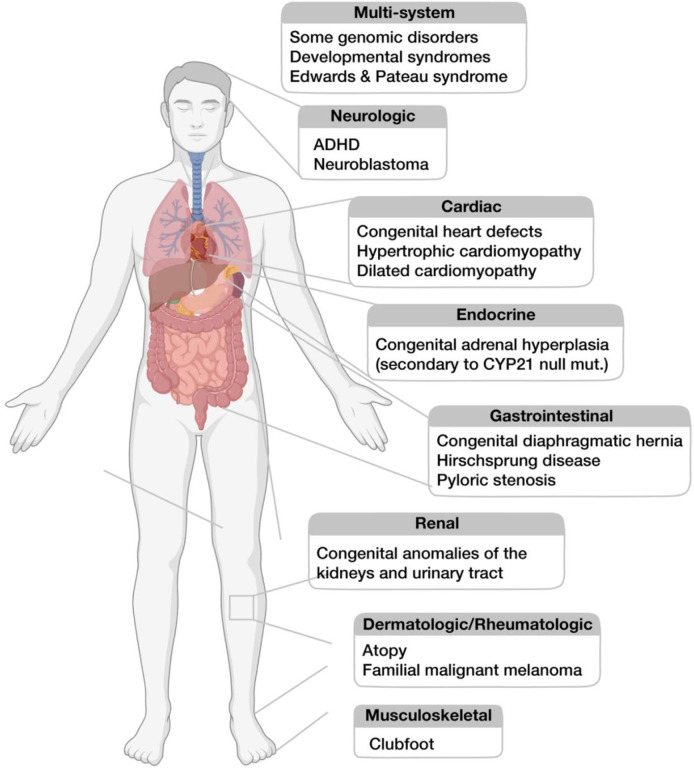
Organ systems affected by male-biased disorders that exhibit an FPE. Associated with Table 2.

We performed a systematic review of 101 genetic studies of male-biased pediatric conditions identified after searching the PubMed and Embase databases, 25 of which satisfied all criteria for inclusion (Fig. 3 and Methods). From these studies, we summarize 16 disorders beyond autism that display an FPE as judged by meeting at least one of the following criteria: (1) epidemiological data supporting the Carter effect; (2) sequencing data demonstrating a greater genetic load of deleterious autosomal variants in affected females compared to males; or (3) male-biased penetrance of a deleterious autosomal variant (Table 2). Only a few of these conditions have been explicitly described as displaying sex-differential liability. They vary in how they impact organ systems (Fig. 4), but they share three characteristics. First, like autism, most are pediatric disorders. Second, many co-occur with autism, and some, such as congenital heart disease, are linked to mutations in known autism risk genes^59^. Finally, they involve disruptions in molecular processes like chromatin modification, transcriptional regulation, or splicing, which are also implicated in autism and, as we have shown, involve Xi-expressed genes (Fig. 2). We therefore propose that female-biased expression of Xi-expressed genes leads to sex differences in ubiquitous molecular processes that drive a broader FPE in other male-biased pediatric conditions.

## Conclusion

**Figure 5.**
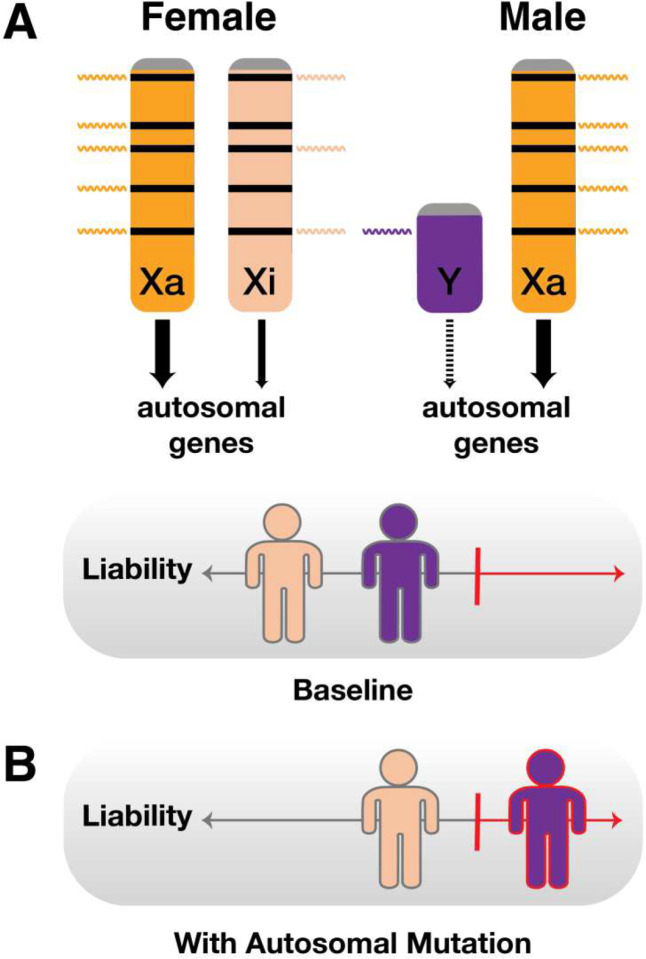
Proposed model by which the human “inactive” X chromosome Xi acts as the female protector. (**A**) The human Xi, found in XX females but not XY males, expresses at least 60 protein-coding genes, including many global regulators of core molecular processes such as chromatin modification. These regulators influence thousands of autosomal genes and are frequently disrupted in autism and other male-biased pediatric disorders. Most of these Xi-expressed genes lack a Y-linked homolog in males. We propose that the female-biased expression of such Xi-expressed genes allows the Xi to act as a protective genetic modifier of autosomal genetic liability for autism and other male-biased disorders in females, (**B**) allowing females to better tolerate deleterious autosomal mutations associated with these conditions compared to males. This model explains the core observation of the female protective effect: females require more autosomal liability than males to reach the liability threshold (red vertical line) necessary to manifest these disorders.

Autism’s pronounced male bias is one of its most striking epidemiological features, and it is well established that females require a greater burden of autosomal genetic risk than males to manifest autism. Here, we propose that this female protective effect arises because of the female-biased expression of Xi-expressed genes that regulate genes and biological processes associated with autism, allowing females to better buffer the effects of autism-associated autosomal mutations. Put another way, we posit that the Xi acts as a protective genetic modifier of autosomal autism liability in females (Fig. 5). This can explain the core observation of the FPE: females can better tolerate *autosomal* autism genetic risk compared to males. Moreover, we demonstrate an FPE in 16 male-biased conditions beyond autism, most of which are pediatric disorders involving disruption to the same core molecular processes implicated in autism and regulated, in part, by Xi-expressed genes. Taken together, we propose that differences in the genetic activities of Xi and Chr Y contribute to higher rates of autism and other pediatric disorders in males, highlighting a general role for the Xi as a female protector in the face of autosomal liability.

## Methods

### Systematic literature review to identify male-biased genetic conditions beyond autism that empirically demonstrate an FPE

An initial set of studies for the literature review was obtained from the electronic biomedical databases PubMed and Embase. A total of 162 studies (61 duplicates) were identified using a combination of an automated search strategy (query: “female protective effect” OR “Carter effect”) and manual curation of genetic studies of known male-biased pediatric disorders. Of the 101 unique studies, 46 were excluded for a primary focus on autism, and 6 were excluded for a primary focus on female-biased disorders (adolescent idiopathic scoliosis and multiple sclerosis) based on abstract review. An additional 24 studies were excluded after full-text review due to insufficient data to assess the presence of a female protective effect (FPE). The remaining 25 studies were reviewed in full for empirical evidence of an FPE based on at least one of the following criteria: (1) epidemiological data supporting the Carter effect; (2) sequencing data demonstrating a greater genetic load of deleterious autosomal variants in affected females compared to males; or (3) male-biased penetrance of a deleterious autosomal variant. This approach identified 16 male-biased disorders beyond autism that exhibit an FPE (Fig. 4 and Table 2). The review pipeline follows PRISMA guidelines and is summarized in Fig. 3.

## Supplementary Files

This is a list of supplementary files associated with this preprint. Click to download.

- TableS109.4.25.xlsx
- SupplementaryMaterial09.4.25.pdf

## Figures and Tables

**Table 1.** Authors’ definitions of terms commonly used in this field.

Term (as applied to autism)	Definition used in this work
**Sex/male bias**	The epidemiological finding that autism is diagnosed more frequently in males than females. The female protective effect (discussed below) contributes to the male bias of autism, as do factors such as underdiagnosis of autistic females and enhanced male susceptibility to X-linked recessive causes of autism.
**The female protective effect (FPE)**	The finding that autistic females, on average, harbor a greater autosomal genetic load for autism as compared to autistic males, suggesting that females exhibit a decreased genetic susceptibility to autism relative to males.
**The sex-differential liability threshold model (SD-LTM)**	The specific conceptualization of the FPE that theorizes that females exhibit a higher liability threshold for autism than males. The difference in the female- and male-specific liability thresholds for autism can be thought of as the FPE. While we focus here on the SD-LTM as applied to autism and other male-biased disorders, the model is generalizable to any sex-biased condition exhibiting multifactorial inheritance.

**Table 2.** Summary of male-biased disorders beyond autism that empirically exhibit an FPE. We consider three types of evidence for the FPE: (1) the Carter effect based on epidemiological data (“familial recurrence data”), (2) the finding that affected females harbor a higher genetic load of deleterious, disorder-associated variants as compared to males from sequencing data of affected individuals (“sequencing data”), and (3) sex-biased penetrance of autosomal genetic variants (“sex-biased penetrance”). Associated with Figure 4.

Condition and Key Citations	Sex Ratio (M:F)	Evidence of FPE		
		Familial recurrence data	Sequencing data	Sex-biased penetrance
**Atopy** ^60^	2:1	Yes		
**Attention-deficit hyperactivity disorder** ^61,62^	2:1	Yes		
**Congenital anomalies of kidney and urinary tract** ^63^	1.44:1			Yes
**Clubfoot** ^56^	1.6–2.5:1	Yes		
**Congenital adrenal hyperplasia, secondary to CYP21 null mutations** ^64^	Only seen in viable females			Yes
**Congenital diaphragmatic hernia** ^65^	1.4:1		Yes	
**Congenital heart disease** ^57,63^	1.5:1	Yes	Yes	
**Dilated cardiomyopathy** ^66^	2:1			Yes
**Familial malignant melanoma** ^67^	2:1	Yes		
**Genomic disorders with extreme phenotypic heterogeneity** ^68^	Varies based on specific genomic disorder; ranges from 1.5:1 to only males affected	Yes		Yes
**Hirschsprung disease** ^69–73^	4:1	Yes	Yes	
**Hypertrophic cardiomyopathy** ^74^	1.5:1			Yes
**Intellectual disability and general developmental disorders** ^13,75^	1.4:1		Yes	
**Neuroblastoma** ^76^	1.3:1			Yes
**Pyloric stenosis** ^24,25^	4:1	Yes		
**Trisomy 13 (Patau syndrome)** **&** **Trisomy 18 (Edwards syndrome)** ^77–79^	1.3: 1 Sex ratio in miscarried fetuses 1.5:1			Yes

## Data Availability

Code and scripts used for the analyses presented in this manuscript are available via GitHub (https://github.com/MayaTalukdar/talukdar-page-xi-female-protector-perspective-analysis).
